# Crossmodal correspondences between visual features and tastes in preschoolers: an exploratory study

**DOI:** 10.3389/fpsyg.2023.1226661

**Published:** 2023-08-14

**Authors:** Xianwei Meng, Na Chen, Junya Ishida, Katsumi Watanabe, Taro Murakami

**Affiliations:** ^1^Graduate School of Informatics, Nagoya University, Nagoya, Japan; ^2^The Gonda Multidisciplinary Brain Research Center, Bar-Ilan University, Ramat Gan, Israel; ^3^Faculty of Education and Care of Early Childhood, Tokoha University, Shizuoka, Japan; ^4^Faculty of Science and Engineering, Waseda University, Tokyo, Japan; ^5^Department of Psychology, University of New South Wales, Sydney, NSW, Australia

**Keywords:** crossmodal correspondence, shape, color, taste, child development, sensory pairing, association

## Abstract

**Introduction:**

Adults possess a natural inclination to associate sensory cues derived from distinct modalities, such as the pairing of sweet with pink. However, studies exploring crossmodal correspondences in children, particularly in the sensory pairing of visual features and tastes, are scant, leaving unanswered questions regarding the developmental trajectory of crossmodal correspondences. The present study investigates whether Japanese preschool children demonstrate specific biases in shape–color, shape–taste, and color–taste associations.

**Methods:**

In a series of in-person experiments, 92 children between 3 to 6 years of age completed matching tasks utilizing paper stimuli.

**Results:**

Children exhibit crossmodal correspondences in shape-color (circle-red and asymmetrical star-yellow), shape–taste (triangle-salty and circle-sweet), and color–taste (yellow-sour, black-bitter, and pink-sweet) associations. Moreover, children’s choices are not influenced by their individual preferences.

**Discussion:**

The crossmodal correspondences observed in this study have been observed in previous research on adults from the same (Japanese) culture, although adults showed more crossmodal correspondences than the children in this study (e.g., pink-circle, triangle-sour, and green-bitter). Thus, while some crossmodal correspondences emerge during childhood, others may require additional time to develop, thereby highlighting the importance of understanding the cognitive mechanisms underlying crossmodal correspondences from an ontogenic perspective.

## 1. Introduction

The cognitive ability to integrate information derived from various features and stimuli dimensions is fundamental in everyday life (Spence, 2011; Stein, 2012). For example, people use the association between size and sound to make various practical decisions: people often link the size of an animal to the sound it produces. Larger animals, like elephants or whales, are associated with deep, booming sounds, while smaller animals like birds are linked to higher-pitched chirping. People also often use the association to assess the size of objects when hearing them drop on the floor. When an object falls and makes a sound upon impact, the sound produced can give us some clues about the object’s size and weight: larger and heavier objects tend to produce louder and lower-pitched sounds when they hit the floor.

Cognitive scientists have investigated the factors or circumstances producing or facilitating such integration to understand how individuals integrate information from different features and dimensions. Traditional research has demonstrated that stimuli are more likely to be associated if presented closely in time and space (Stein et al., 1988). Additionally, individuals tend to associate information when their meanings are related rather than unrelated (e.g., pairing a woofing sound with a static image of a dog) (Molholm et al., 2004; Hein et al., 2007). Recently, associations have been identified among fundamental stimulus characteristics across various modalities, such as color, shape, taste, pitch, size, and brightness. These are shared by many individuals, and some of these seem to be culture-independent (e.g., black color-bitter taste; Wan et al., 2014) or have deep developmental origins [e.g., the low pitch-big size association observed in 3-month-old infants; (Pietraszewski et al., 2017)]. These mappings are commonly referred to as “crossmodal correspondences” (Spence, 2011; Parise and Spence, 2014).

Crossmodal correspondence has been explained through four distinct categories of accounts: emotional, semantic, statistical, and structural. The first posits that correspondence arises because unimodal stimuli elicit similar emotional experiences, providing a plausible explanation for certain color–taste correspondences, such as pink-sweet and black-bitter, mediated by positive and negative emotions, respectively (Velasco et al., 2016; Spence, 2020a; Di Stefano et al., 2021; Spence and Di Stefano, 2022). By contrast, the second asserts that correspondence arises from the shared meaning of stimuli. For example, in some languages, pitch is described using the spatial terms “thin and thick,” and these labels can promote crossmodal mappings (Shayan et al., 2014). This account may also elucidate the way we associate animal appearances with voices (Molholm et al., 2004; Hein et al., 2007). According to the third, correspondence results from statistical learning through exposure to co-occurring stimuli in an environment. For example, the crossmodal correspondence between auditory pitch and spatial elevation (e.g., high or low sounds) has been considered to be extracted from experience; when analyzing natural sounds from the environment, researchers found a clear mapping between frequency and elevation (i.e., higher-pitched sounds occur more frequently in higher space; Parise et al., 2014). This account also enables us to understand the reason why some crossmodal correspondences (e.g., correspondences between certain colors/shapes and tastes) vary between cultures (Wan et al., 2014). Finally, the fourth posits that correspondence arises because of unimodal stimuli sharing the same or interconnected neural basis, which may be innate (Wagner and Dobkins, 2011; Deroy and Spence, 2013; Ludwig and Simner, 2013; Pietraszewski et al., 2017). Wagner and Dobkins (2011) demonstrated that certain shapes influence color preferences in typical 2- and 3-month-olds, but not in 8-month-olds or adults, suggesting that exuberant neural connectivity facilitates synesthetic associations during infancy. These categories of crossmodal correspondences are not mutually exclusive and often overlap, making it challenging to explain them using a single hypothesis.

Although a multitude of empirical studies have investigated crossmodal correspondence, the majority have solely focused on adult participants (Wan et al., 2014; Chen et al., 2021). Consequently, there exists a gap in the literature regarding the developmental trajectory of crossmodal correspondences, particularly related to the sensory pairing of visual features (color/shape) and tastes (for developmental studies of other correspondences, see Simpson et al., 1956; Lewkowicz and Turkewitz, 1980; Ludwig and Simner, 2013; Pejovic and Molnar, 2017; Thomas et al., 2017; Chow et al., 2021; Speed et al., 2021). Previous research has found adults exhibiting specific color–taste/shape–taste crossmodal correspondences, such as yellow–sour, red–sweet, angular–sour/bitter, and round–sweet associations (Spence and Gallace, 2011; Spence and Ngo, 2012; Spence, 2012, 2019; Bremner et al., 2013; Wan et al., 2014; Spence et al., 2015; Velasco et al., 2015; Saluja and Stevenson, 2018; Spence and Levitan, 2021). However, literature on younger participants, such as preschool children, is lacking. Studying preschoolers can illuminate whether these crossmodal associations occur independent of the written language and understanding of metaphorical uses of language, and limited life experiences, as compared to adults (Spector and Maurer, 2009, 2011; Meng et al., 2022). Moreover, testing toddlers over infants is advantageous because they understand simple verbal instructions and can be tested using methods yielding easily interpreted data from more test points (Spector and Maurer, 2009).

Previous studies suggest that learning that involves acquisition of associations between environmental stimuli, may account for a certain crossmodal correspondence. For instance, infants as young as 4 months of age have demonstrated the ability to learn arbitrary bimodal correspondences between color and taste, as evidenced by their consistent selection of the color that had been paired with sweetness (Reardon and Bushnell, 1988). Moreover, cross-cultural variations have been observed in the correspondence between specific colors, shapes, and taste terms, indicating that adults’ associations may stem from their daily experiences (Wan et al., 2014). However, no study has provided robust evidence on whether children exhibit adult-like color–taste/shape–taste crossmodal correspondences. To date, only Fateminia et al. (2020) have investigated the association between color and taste in preschool children. The study involved presenting a group of Iranian preschoolers, aged 2–6 years, with eleven colors categorized as primary and secondary, and asking them to select a taste corresponding to each color from a set of options, including sour, salty, sweet, bitter, pungent, astringent, and tasteless. Although the authors interpreted the results as revealing “meaningful and clear relationships” between colors and tastes, the data analysis relied solely on descriptive statistics, failing to investigate the likelihood that the outcomes were due to chance or actual effects, and whether they could be generalized to a wider population (Fateminia et al., 2020).

To bridge this gap, the present study investigates the crossmodal correspondences between visual features and tastes in preschool-aged children. The selection of this age group has enabled the utilization of more sophisticated experimental methods such as manual or verbal responses, typically employed in adult studies, while ensuring that the children possessed the cognitive capacity to comprehend and complete the tasks. To ensure comparability with previous research conducted on adult populations, we modeled our study design after the work of Chen et al. (2021), who investigated shape–color, shape–taste, and color–taste correspondences in Japanese adults. The tasks were modified to be more age-appropriate for children (Chen et al., 2021).

In a series of in-person experiments, children engaged in matching tasks using paper stimuli. The stimuli involved the following elements. (1) Five shapes: a triangle, circle, square, blob (bouba), and an asymmetric star (kiki) (Köhler, 1947; Chow and Ciaramitaro, 2019); (2) nine colors: black, blue, green, orange, pink, purple, red, white, and yellow; and (3) four basic taste words: sweet, sour, bitter, and salty (Spence and Levitan, 2021). This is the first study to investigate crossmodal associations between visual features and taste in preschool-aged children by examining three different types of sensory pairings. We conducted this research with an exploratory approach because we did not formulate any specific hypotheses regarding the potential correspondence patterns that might have emerged from the participating children.

## 2. Method

### 2.1. Participants

The final sample included 92 3- to 6-year-old children living in Kitakyushu, Japan (50 girls and 42 boys; mean age = 62.21 months, SD = ±9.83). We determined our sample size following Chen et al. (2021) who investigated crossmodal correspondences in adults using similar tasks. One 3-year-old child did not provide answers to some of the questions (regarding the taste of purple, potato, strawberry, and green pepper); the child’s data from the provided answers was nonetheless included in the analysis. Prior written consent was obtained from the children’s caregivers. This study was approved by the Ethics Committee of Tokoha University (No. 22-20) and conducted in accordance with the ethical standards of the American Psychological Association (2017) and the Declaration of Helsinki (2001).

### 2.2. Set-up

The experiment was conducted in a quiet room at a kindergarten. Three researchers individually tested each child. Each pair of children and the experimenter sat face-to-face across a child’s table. The experimenter presented the paper stimuli on the table and asked the children questions. All experimental sessions were video recorded.

### 2.3. Materials

**Figure 1:**
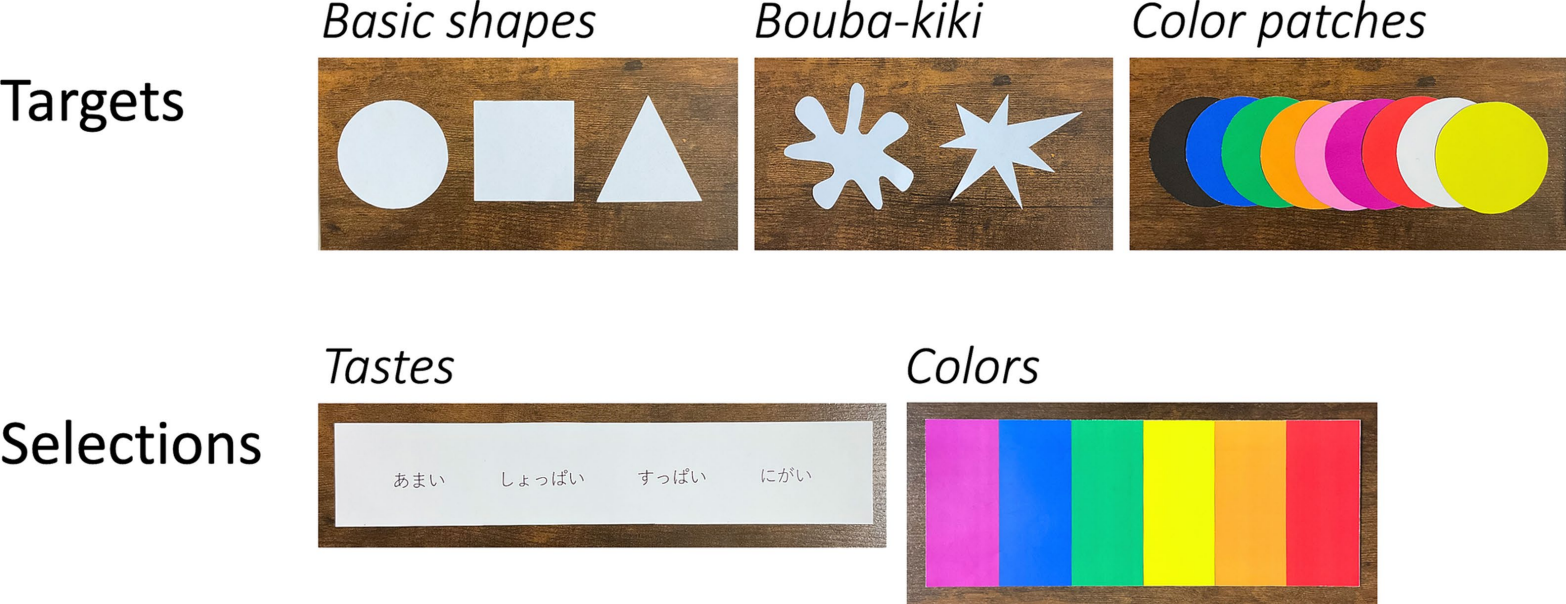
Materials of the experiment.

All stimuli were created using PowerPoint (Microsoft) and printed onto white hard paper. Nine color paper patches and five geometric shapes were used as “target” stimuli (Chen et al., 2021; Figure 1). The patches were circular with a diameter of 5.5 cm and were black, blue, green, orange, pink, purple, red, white, and yellow in color. The geometric shapes included circle, square, triangle, asymmetrical star, and blob, and the sizes were similar to the color patch size.

Four basic taste terms and six colors were used as “selection” stimuli. The taste items were presented in black Japanese text as “にがい (bitter),” “しょっぱい (salty),” “すっぱい (sour),” and “あまい (sweet).” The colors selected from the target stimuli were blue, green, orange, purple, red, and yellow. All colors were printed on a piece of paper. The order of the taste items and colors from left to right was created in two patterns, and each participant was randomly assigned to either pattern.

### 2.4. Procedure

The experiment consisted of three phases: warm-up, crossmodal correspondence, and follow-up. Prior to the experiment, the researcher explained to each child that they would participate in an interesting quiz game. All participants categorically affirmed that they wanted to play the game.

The warm-up phase familiarized the children with the experimental circumstances and materials. The researcher showed the children the target color patches and asked them to indicate the names of the colors. Then, they were presented with two black-and-white pictures: a girl and a boy cartoon characters. For each character, children were asked to choose a color from the color selections for their t-shirts. The pictures were presented randomly.

The crossmodal correspondence phase consisted of two sessions in a fixed order: the basic shape and bouba–kiki, in which circles, squares, and triangles and asymmetrical stars and blobs, respectively, were used as the target shapes. Each session consisted of a shape–color matching task followed by a shape–taste matching task. In the first task, the target shapes were presented individually at the center of the table, and the participants were asked to choose a color from the color selections that best matched each shape. Whereas, in the second task, they had to choose a taste from the taste selections that best matched the target shapes. To ensure that children understood the words for the tastes presented as selections, the researcher read each word (e.g., “This word is *sweet*”) and confirmed that children could match each word on the paper with what they read. Additionally, a color–taste matching task was conducted between the two sessions, in which participants were shown the target color patches and asked to choose a taste from the taste selection. In each matching task, the target stimuli were presented in random orders.

The follow-up phase had two aims. The first was to test whether the participants understood the concept of taste. Therefore, in the taste-comprehension task, they were shown pictures of French fries, green peppers, lemons, and strawberries and asked to choose a taste from the taste selections that best matched each food. These foods were selected assuming that they are typical foods recognized by children as salty, bitter, sour, and sweet. The second aim was to test whether the children’s color choices were influenced by their preferences. Therefore, a color-preference task was conducted where participants were asked to choose one color that they liked best from the color selection.

### 2.5. Analysis plan

Chi-square tests were used to determine whether the children showed crossmodal correspondences. For each matching test, we examined whether the choices varied by category. Furthermore, an adjusted residual analysis was used to determine which choice was significantly associated with a certain target (i.e., color–taste/shape–taste/shape–color associations). The analysis shows which selection is chosen more frequently than expected by chance for each target. Positive and negative residual values indicate more and less frequently chosen than expected, respectively; here, only positive residuals were used (Agresti, 2012; Chen et al., 2015a). A similar analysis was conducted for the taste-comprehension task. Furthermore, the correlation coefficient between the children’s judgment in the crossmodal correspondence phase and the color preference task was evaluated.

## 3. Results

### 3.1. Basic shapes session (shape–color and shape–taste matching tasks)

**Figure 2:**
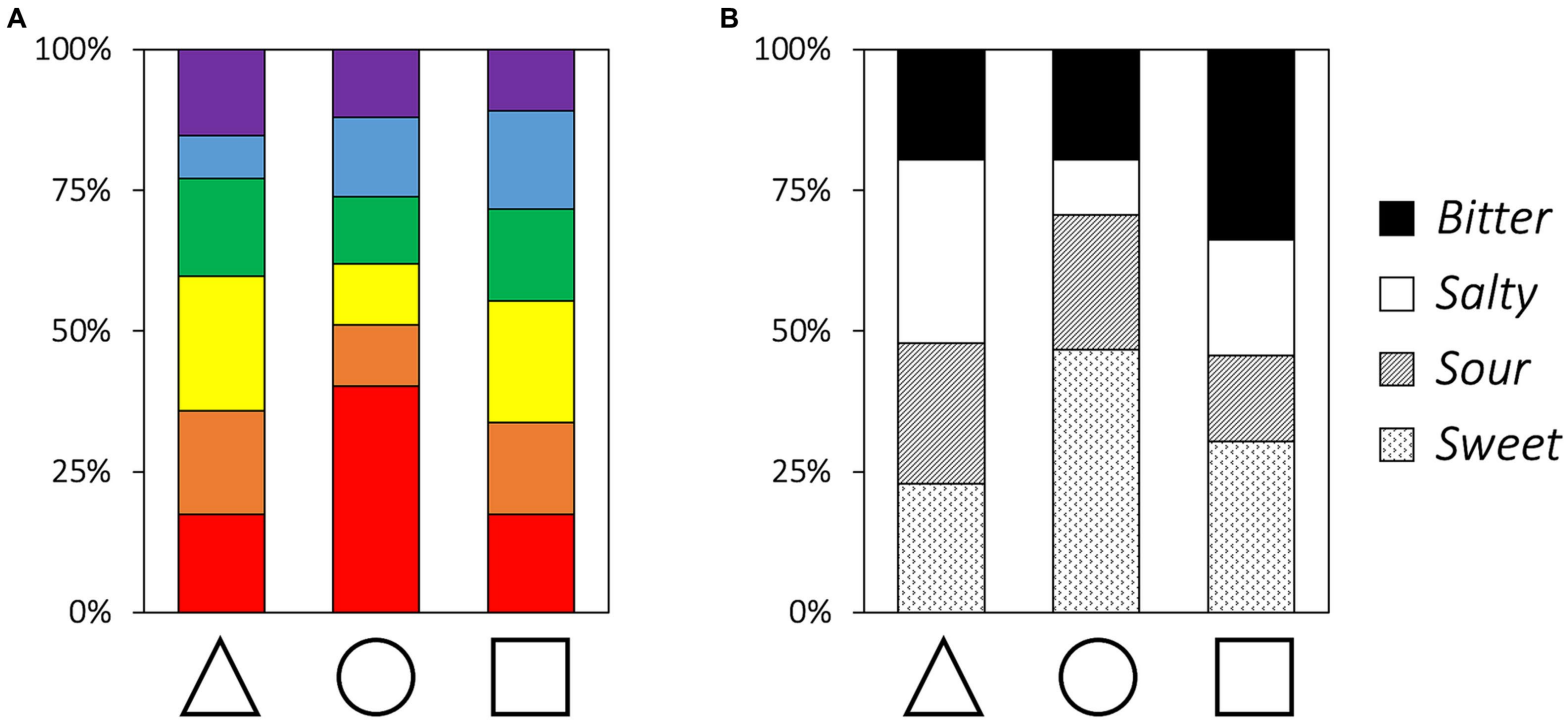
Proportions of color **(A)** and taste **(B)** choices for each basic shape.

The results of the Chi-square test show that the color choices vary by the shape categories (*χ*^2^ = 24.65, *df* = 10, *p* = 0.006, Cramer’s *V* = 0.211; Figure 2). The adjusted residual results show the association of a circle with red color (z = 4.13, adjusted *p* = 0.001, *p*-values are corrected using Holm’s method for multiple comparisons). Furthermore, the taste choices vary by shape categories (*χ*^2^ = 27.17, *df* = 6, *p* < 0.001, Cramer’s *V* = 0.222). The adjusted residual results indicate the association of the triangle with salty (z = 3.34, adjusted *p* = 0.010), and circle with sweet taste (*z* = 3.34, adjusted *p* = 0.010), respectively.

### 3.2. Color–taste matching task

**Figure 3:**
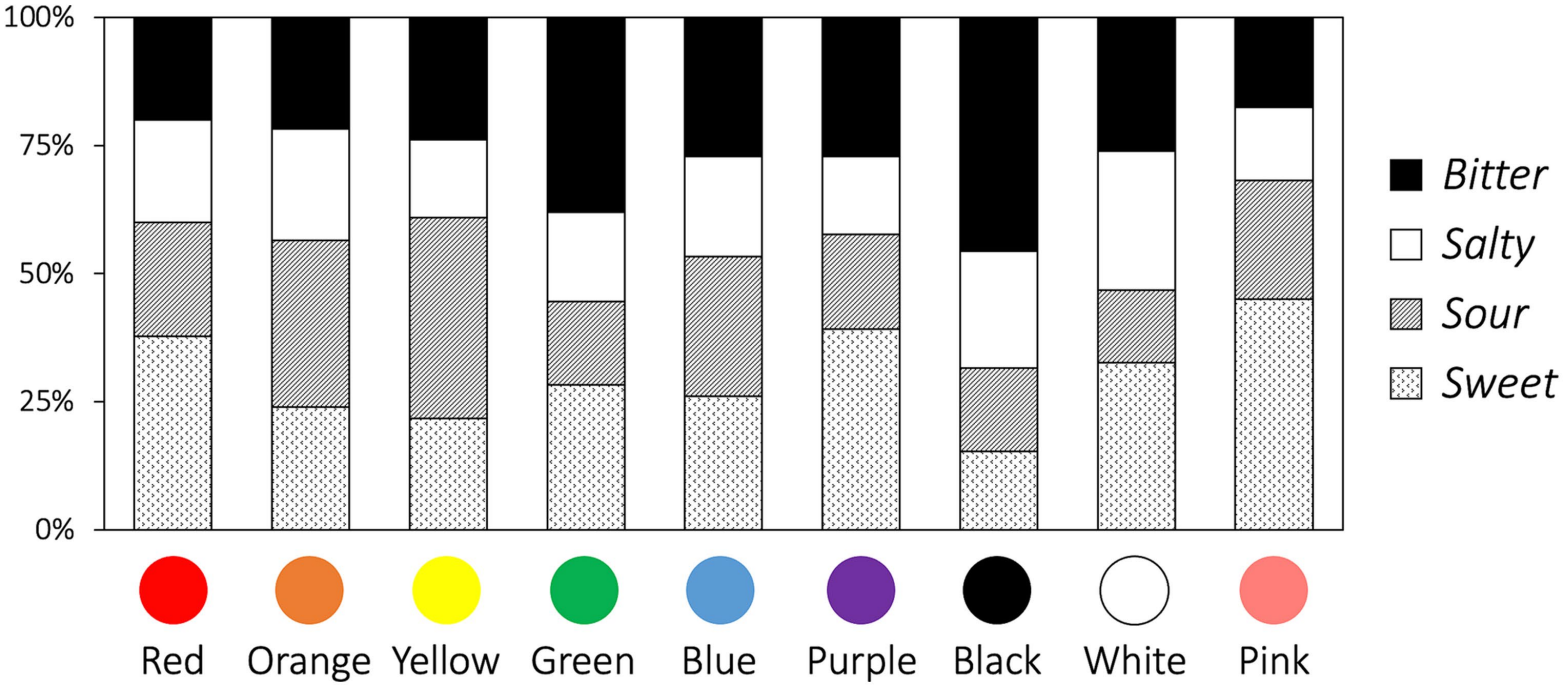
Proportion of taste choices for each color.

The results of the Chi-square test show that taste choices vary by color categories (*χ*^2^ = 72.33, *df* = 24, *p* < 0.001, Cramer’s *V* = 0.171; Figure 3). Specifically, yellow, black, and pink are associated with sour (*z* = 3.82, adjusted *p* = 0.005), bitter (*z* = 4.13, adjusted *p* = 0.001), and sweet taste (*z* = 3.34, adjusted *p* = 0.029), respectively.

### 3.3. Bouba–kiki session (shape–color and shape–taste matching tasks)

**Figure 4:**
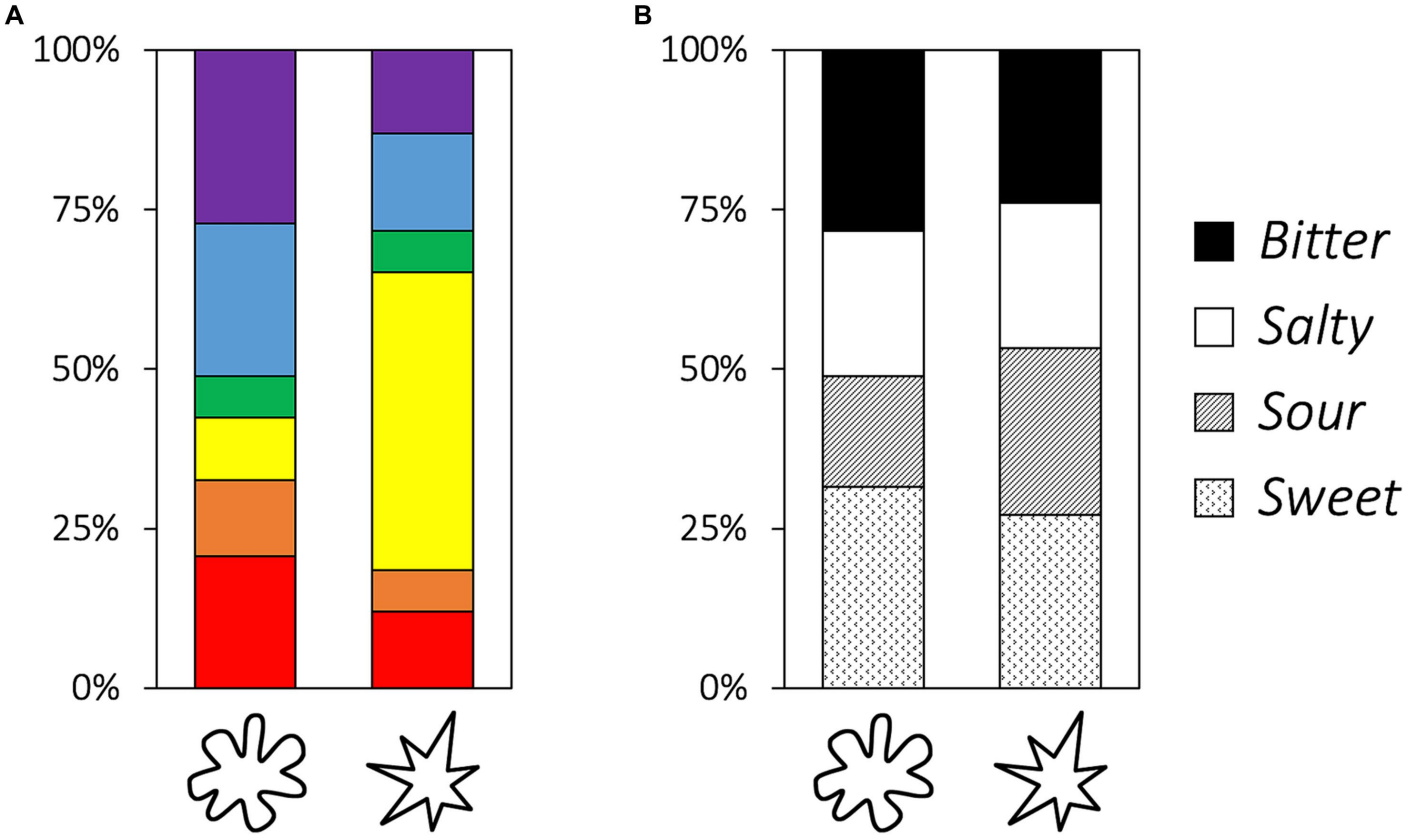
Proportion of color **(A)** and taste **(B)** choices for blob and asymmetrical star.

The results of the chi-square test show that color choices vary according to the shape categories of the bouba–kiki session (*χ*^2^ = 32.18, *df* = 5, *p* < 0.001, Cramer’s *V* = 0.418; Figure 4); however, there is no significant association between taste and shape (χ2 = 2.23, df = 3, *p* = 0.526, Cramer’s *V* = 0.110). The adjusted residual results show that a kiki is associated with yellow (z = 5.57, adjusted *p* = < 0.001).

### 3.4. Follow-up

**Figure 5:**
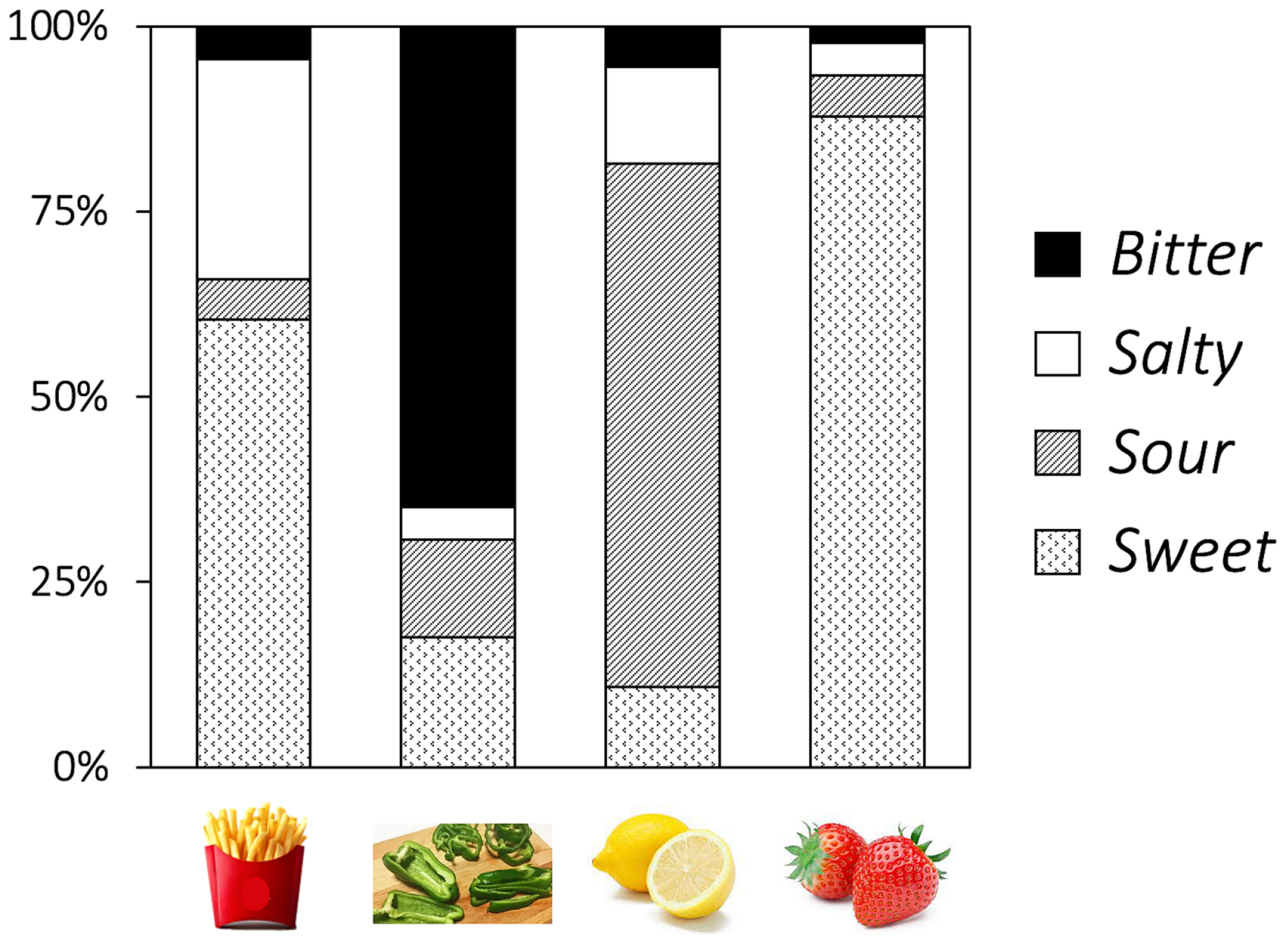
Proportion of taste choices for French fries, green pepper, lemon, and strawberry.

For the taste-comprehension task, the results of the Chi-square test show that taste choices vary by foods (*χ*^2^ = 395.38, *df* = 9, *p* < 0.001, Cramer’s *V* = 0.573; Figure 5). The adjusted residual results show that children associate French fries with salty (z = 5.52, adjusted *p* < 0.001) and sweet taste (*z* = 3.62, adjusted *p* = 0.001). Furthermore, they associate green pepper with bitter (*z* = 12.77, adjusted *p* = < 0.001), lemons with sour (*z* = 12.19, adjusted *p* = < 0.001), and strawberries with sweet taste (*z* = 9.71, adjusted *p* = < 0.001), respectively.

Correlation statistics indicate that children’s color choices in crossmodal correspondence tasks inadequately agree with their color preferences (McHugh, 2012). The kappa coefficients of the paired data between color preference and color choices for triangles, circles, squares, blobs, and asymmetrical stars were 0.032, 0.041, 0.023, 0.172, and −0.020, respectively.

## 4. Discussion

Previous studies indicate that adults consistently associate features of different sensory modalities, such as sweet taste with pink colors. To better understand the mechanisms underlying the acquisition of crossmodal correspondence, this study explores crossmodal associations between visual features and taste in preschoolers using paper-based stimuli. The participants were asked to match color to shape, taste to shape, and taste to color. The results indicate that children associate red with circles and yellow with asymmetric stars, salty with triangles and sweet with circles, and sour with yellow, bitter with black, and sweet with pink in shape–color, shape–taste, and color–taste tasks, respectively.

Several shape–color correspondences observed in the current study sample of children differed from those found in previous studies involving adult participants who shared the same cultural background (Chen et al., 2021). According to Chen et al. (2021), adults tend to associate red and pink with circles, yellow with triangles and asymmetric stars, blue with squares, and green with blobs. The pink–circle, yellow–triangle, blue–square, and green–blob associations were not found in the current sample. Note that the red–circle and yellow–asymmetrical star associations might be the result of exposure to frequent combination occurrences in the environment, such as the Japanese flag and night sky stars (Chen et al., 2015a,b). Therefore, even though some color–shape associations might be observed from early infancy (Wagner and Dobkins, 2011), the current findings may provide evidence supporting the hypothesis that shape–color correspondences are the result of statistical learning from environmental co-occurrences (Parise et al., 2014). One plausible explanation for the absence of a yellow–triangle association is that the yellow–asymmetrical star association is acquired earlier, and then generalized to a yellow–triangle association during development, given that both shapes have sharp angles. In line with the learning hypothesis, studies have demonstrated that even the most basic shapes used in this study, such as circles, squares, and triangles, exhibit cultural variations in their shape–color correspondences; for instance, German participants showed red–triangle, blue–square and yellow–circle association correspondences, whereas Japanese participants showed yellow–triangle, blue–square and red–circle association correspondences (Jacobsen, 2002; Chen et al., 2015a; Dreksler and Spence, 2019). In another stream of research, Spector and Maurer (2008, 2009, 2011) explored the associations between alphabets and colors in pre-literate and literate children and adults in Canada. Their findings revealed that the participants in all groups associate specific letters with colors (e.g., O with white and X with black), presumably based on their shapes but not their phonetic or morphological properties (e.g., B with blue). Furthermore, preliterate children did not exhibit the same associations observed in literate children and adults (e.g., A with red and G with green). These results, combined with the ones of the present study, suggest that even pre-literate children’s shape–color correspondence is influenced by cultural factors (e.g., circle is associated with red in Japanese children and with white in Canadian children), consistent with the learning hypothesis.

The participating children also exhibited several shape–taste correspondences: associating salty with triangles and sweet with circles. This finding is consistent with that of previous research on adults in Japanese culture, where sharp-angled (e.g., triangles, asymmetrical stars), circular (e.g., circles), right-angled (e.g., squares), and blob shapes are associated with sour, sweet, salty, and bitter taste, respectively (Chen et al., 2021). Notably, our follow-up taste comprehension task confirmed that participating children could accurately differentiate between salty and sour tastes, thus supporting the significance of the observed salty-triangle association. A developmental shift in shape–taste correspondence may occur from preschoolers to adults, whereby the association between salty taste and triangles shifts to sour taste and triangles. Several mechanisms have been proposed to account for the development of shape–taste correspondences, including shape–taste experiences [e.g., sweet candies being typically round in shape; Spence, 2023; see also Speed et al. (2021)‘s notion on the perspective of odor–shape association] and emotional mediation (e.g., the association between sweet taste and roundness owing to their positive valence; Blazhenkova and Kumar, 2018). Future research should investigate the potential role of these mechanisms in shaping shape–taste correspondences in children.

The present findings on color–taste correspondence in children are consistent with those of previous studies conducted on adults across various cultures (O’mahony, 1983; Koch and Koch, 2003; Tomasik-Krótki and Strojny, 2008; Wan et al., 2014; Spence et al., 2015; Woods et al., 2016; Woods and Spence, 2016; Spence, 2019; Chen et al., 2021). For adult participants sharing the same cultural background as that of the children in our study, Chen et al. (2021) observed additional color–taste associations, specifically orange with sour, blue and white with salty, and green and purple with bitter. Based on our follow-up taste-comprehension task results, which indicated that the participants were able to correctly identify sour, salty, and bitter tastes, the observed color–taste correspondences may reflect a developmental change (Chen et al., 2021). A green–bitter association has been documented in adults across different cultures (Saluja and Stevenson, 2018; Chen et al., 2021). Interestingly, although the children also associated bitter taste with green peppers, they did not exhibit a significant bias toward linking bitter taste with green. Adult participants also often associate green with sourness (O’mahony, 1983; Koch and Koch, 2003; Tomasik-Krótki and Strojny, 2008; Wan et al., 2014; Woods et al., 2016; Woods and Spence, 2016), which may be because of their experience consuming sour green fruits. Similar associative learning mechanisms may account for the development of the white–salty association, which may be established through exposure to salt. Our results indicate that children judged French fries as not only salty but also sweet. This may be because children are prevented from consuming excessive amounts of salt in their daily diet, including that coming from French fries, for health-related reasons. Another reason might be that the presence of added sugar in certain industrial fries could contribute to the perception of sweetness in the context of French fries (Atsumi et al., 2023).

Regarding previous findings on color–taste correspondences in children having different cultural backgrounds, the descriptive statistics of Fateminia et al. (2020) showed that 2- to 6-year-old Iranian children tend to associate blue with sour, red with sweet and sour, yellow with sweet and sour, purple with bitter, orange with sour, green with sour, black with bitter, white with sweet, pink with sweet (Fateminia et al., 2020). Some of these correspondences (i.e., yellow–sour, black–bitter, and pink–sweet), but not others, are shared by the current study sample of Japanese children. These imply that the acquisition of color–taste correspondences may be both culture-dependent and culture-independent, depending on the nature of certain correspondences.

Nevertheless, the current experimental evidence supports the notion that experience-based explanations may underlie color–taste associations. Higgins and Hayes (2019) demonstrated using a repeated brief exposure paradigm that color–taste associations may be learned through repeated exposure, even when the exposure period is relatively brief (e.g., several days).

Contrastingly, even though the participating children associated green pepper with bitter taste, their association with it was black, rather than green. Moreover, young children may have limited opportunities to be exposed to a bitter taste. These findings and considerations suggest that alternative mechanisms for the acquisition of color–taste correspondence in children must be considered. One such mechanism may be emotional or hedonic associations, as previous research has shown that bitter taste is generally disliked or associated with danger (Higgins and Hayes, 2019; Spence, 2023). Additionally, black is the least favorite color of children and elicits negative emotions (Boyatzis and Varghese, 1994; Koleoso et al., 2014). Therefore, it is plausible that the association between bitter taste and black color in children is based on shared negative values (Salgado-Montejo et al., 2015; Velasco et al., 2015; Turoman et al., 2018; Spence, 2023). Future research should use more detailed measures, such as taste and color evaluation, to directly test this hypothesis.

This study has several limitations which raise important questions that can form the basis for future research. First, to make the tasks more child-friendly, we limited the shapes to basic geometric shapes and the Bouba–Kiki pair (Chow and Ciaramitaro, 2019). Additionally, we limited the tastes to four basic tastes (sweet, sour, bitter, and salty), although many food scientists suggest that there are more than 20 basic tastes (Stuckey, 2012). To explore other crossmodal correspondences, future studies should include a broader range of stimuli, while ensuring that children make reliable choices.

Second, children were asked to select colors for shapes, tastes for shapes, and tastes for colors. These selections are unidirectional, making it unclear whether children link items bidirectionally (Wan et al., 2014; Chen et al., 2021). For instance, when asked to choose a taste for yellow color, children may first imagine yellow food, such as lemons, and select sour for yellow because lemons have a sour taste. However, if the procedure was reversed and children were asked to choose a color for sour taste, whether a yellow–sour correspondence would still occur cannot be said (Fateminia et al., 2020). Some crossmodal correspondences have been thought to be bidirectional; for instance, larger objects are matched with lower-pitched sounds, and lower-pitched sounds are just as strongly associated with larger objects (Deroy and Spence, 2013; Spence, 2020b). However, this does not mean that all crossmodal correspondences are bidirectional. Future studies need to investigate the directionality of cross-modal correspondences in children, as it contributes to our understanding of the precise nature of the relationships between associated stimuli (Posner et al., 1976; Spence et al., 2001; Spence, 2019). Collaterally, future studies could also investigate whether individuals’ crossmodal correspondences show consistency across different sensory modalities (e.g., a yellow–triangle–sour correspondence), and whether and how such tendencies vary between individuals. Chen et al. (2021) found that Japanese adult participants show certain color–taste and shape–color associations and that such associations were observed more frequently in individuals with lower autistic quotient scores. This was in line with the notion that individuals with autism tend to have a decreased ability to perform statistical learning with co-occurrences and regularities in the environment, leading to difficulties in associating and integrating sensory information across modalities. Therefore, one hypothesis might be that one crossmodal correspondence (e.g., color–taste association) could be predicted based on other crossmodal correspondences (e.g., taste–shape and color–shape associations), especially for individuals who have a higher ability to perform statistical learning from the environment.

Third, the current study had a limited sample size and included a wide range of ages, which precluded testing for potential developmental changes in the observed crossmodal correspondences in the participating children. While the preschoolers in this study can be classified as preliterate, their experiences with daily visual features and tastes, as well as their cognitive abilities to complete tasks, undergo significant developmental changes during the preschool period. Moreover, because the current study was the first attempt to test crossmodal associations between visual features and taste in Japanese preschoolers, there was no information on effect size that could be used for the sample size calculation. Therefore, we followed the sample size applied in the Japanese adult study (Chen et al., 2021). As the current findings revealed relatively small effect sizes of the crossmodal correspondences, future studies need larger sample sizes to enhance the power of the tests in children. Taken together, larger-scale studies are needed to investigate the developmental trajectory of crossmodal correspondence in children.

The present study reveals that children exhibit fewer and more broadly distributed crossmodal correspondences compared to those observed in adults coming from different cultural backgrounds, supporting the notion that the cross-modal associations between visual features and tastes are learned from environmental co-occurrences (Spector and Maurer, 2008, 2009, 2011; Deroy and Spence, 2016; Higgins and Hayes, 2019; Spence and Levitan, 2021). Nonetheless, certain correspondences, such as the black–bitter association, may be better explained by other basic mechanisms. These results represent a new wave of developmental research aimed at elucidating how individuals map and integrate information based on various features and dimensions.

## Data availability statement

The raw data supporting the conclusions of this article will be made available by the authors, without undue reservation.

## Ethics statement

The studies involving humans were approved by the Ethics Committee of Tokoha University (no. 22-20). The studies were conducted in accordance with the local legislation and institutional requirements. Written informed consent for participation in this study was provided by the participants’ legal guardians/next of kin.

## Author contributions

XM, NC, JI, KW, and TM designed the study. XM created the stimuli. XM, JI, and TM conducted the experiments. XM analyzed the data and drafted the manuscript. All authors contributed to the article and approved the submitted version.

## Funding

This work was supported by the JSPS grants-in-aid for Scientific Research (20K20156, 20K14176, 23K02873, 23H03702, and 22H00090).

## Conflict of interest

The authors declare that the research was conducted in the absence of any commercial or financial relationships that could be construed as a potential conflict of interest.

## Publisher’s note

All claims expressed in this article are solely those of the authors and do not necessarily represent those of their affiliated organizations, or those of the publisher, the editors and the reviewers. Any product that may be evaluated in this article, or claim that may be made by its manufacturer, is not guaranteed or endorsed by the publisher.
